# The Influence of Long-Term Treatment with Asenapine on Liver Cytochrome P450 Expression and Activity in the Rat. The Involvement of Different Mechanisms

**DOI:** 10.3390/ph14070629

**Published:** 2021-06-29

**Authors:** Przemysław J. Danek, Ewa Bromek, Władysława A. Daniel

**Affiliations:** Department of Pharmacokinetics and Drug Metabolism, Maj Institute of Pharmacology, Polish Academy of Sciences, Smętna 12, 31-343 Kraków, Poland; danek@if-pan.krakow.pl (P.J.D.); bromek@if-pan.krakow.pl (E.B.)

**Keywords:** asenapine, chronic treatment, rat liver, cytochrome P450 expression, enzyme activity

## Abstract

Therapy of schizophrenia requires long-term treatment with a relevant antipsychotic drug to achieve a therapeutic effect. The aim of the present study was to investigate the influence of prolonged treatment with the atypical neuroleptic asenapine on the expression and activity of rat cytochrome P450 (CYP) in the liver. The experiment was carried out on male Wistar rats. Asenapine (0.3 mg/kg s.c.) was administered for two weeks. The levels of CYP mRNA protein and activity were determined in the liver and hormone concentrations were measured in the pituitary gland and blood serum. Asenapine significantly decreased the activity of CYP1A (caffeine 8-hydroxylation and 3-N-demethylation), CYP2B, CYP2C11 and CYP3A (testosterone hydroxylation at positions 16β; 2α and 16α; 2β and 6β, respectively). The neuroleptic did not affect the activity of CYP2A (testosterone 7α-hydroxylation), CYP2C6 (warfarin 7-hydroxylation) and CYP2E1 (chlorzoxazone 6-hydroxylation). The mRNA and protein levels of CYP1A2, CYP2B1, CYP2C11 and CYP3A1 were decreased, while those of CYP2B2 and CYP3A2 were not changed. Simultaneously, pituitary level of growth hormone-releasing hormone and serum concentrations of growth hormone and corticosterone were reduced, while that of triiodothyronine was enhanced. In conclusion, chronic treatment with asenapine down-regulates liver cytochrome P450 enzymes, which involves neuroendocrine mechanisms. Thus, chronic asenapine treatment may slow the metabolism of CYP1A, CYP2B, CYP2C11 and CYP3A substrates (steroids and drugs). Since asenapine is metabolized by CYP1A and CYP3A, the neuroleptic may inhibit its own metabolism, therefore, the plasma concentration of asenapine in patients after prolonged treatment may be higher than expected based on a single dose.

## 1. Introduction

Cytochrome P450 (CYP) enzymes are members of the superfamily of heme-containing monooxygenases that catalyze the metabolism of endogenous substances (e.g., steroids, monoaminergic neurotransmitters, arachidonic acid, bile acids) and the majority of clinically important drugs including psychotropics (neuroleptics, antidepressants, anxiolytics) [1,2]. The CYP enzymes are located primarily in the endoplasmic reticulum of hepatic cells. Among various CYP enzymes, CYP1, CYP2 and CYP3 are three major families that are involved in the metabolism of approximately 90% of drugs on the market [2]. Drugs that can inhibit or induce the metabolic activity of the CYP enzymes have the potential to affect the metabolism of concomitantly administered drugs, and to lead to changes in their efficacy or to increase the risk of adverse effects [3,4,5,6]. The non-selective mechanism-based CYP inhibitors, such as 1-aminobenzotriazole or atipamezole (pan-specific), are useful tools for discovering CYP-governed metabolism of newly projected drugs or pharmacological tools, and for showing the influence of the enzyme on drug pharmacokinetics [7,8,9,10,11]. On the other hand, modern predictions of CYP-mediated sites of drug metabolism using advanced computer programs enable the modulation of the metabolism of potential drugs to improve their safety and efficacy during the early stages of drug discovery [12].

Our recent studies have provided evidence for an important role of the brain monoaminergic systems, including dopaminergic [13], noradrenergic [14,15] and serotonergic [16] routes, in the physiological neuroendocrine regulation of cytochrome P450 expression in the liver. CYP enzymes are under hormonal control, involving growth hormone, cortisol/corticosterone, thyroid hormones and sex hormones, which are governed by the nervous system [17]. By acting via membrane, cytoplasmic or nuclear receptors, those hormones regulate the transcription of CYP genes, affecting the expression and activity of particular CYP enzymes. Moreover, the nervous system can affect immunological responses, thereby influencing the level of cytokines, which are also regulators of CYP gene expression [17]. Thus, changes in brain neurotransmission may influence cytochrome P450 expression via neuroendocrine or neuroimmune mechanisms. Those central mechanisms may also be involved in the enzyme regulation by psychotropic drugs [16,17,18].

Schizophrenia is a widespread and complex psychiatric disorder, characterized by positive and negative symptoms including psychotic disturbances engaging dopaminergic mesolimbic pathway and emotional withdrawal involving dopaminergic mesocortical pathway, respectively [19,20,21]. Basic symptoms, such as subjective disturbances of self-experience and awareness affect self–other relationship. Self-experience deficits are reflected by disordered cognition, perception and emotion as a result of aberrant neural functional connectivity [22,23]. Asenapine is a novel atypical antipsychotic drug approved for the treatment of schizophrenia, ameliorating both positive and negative symptoms [24,25,26,27]. Asenapine has been also recommended for the treatment of manic/mixed episodes with or without psychotic features associated with bipolar I disorder, such as manic-depressive bipolar disorder [28,29,30]. As asenapine is a tetracyclic member of the dibenzooxepinopyrroles, derived from the tetracyclic antidepressant mianserin, it would be expected to have also an antidepressant effect [31,32]. Asenapine’s mechanism of action is mainly mediated by 5-HT_2A_ and D_2_ receptor antagonism. In addition, it has a potent antagonistic effect on the serotonergic receptor subtypes 5-HT_2B_, 5-HT_2C_, 5-HT_6_ and 5-HT_7_, the adrenergic receptor subtypes α_1A_, α_2A_, α_2B_, α_2C_ and the dopaminergic receptor subtypes D_3_ and D_4_. Moreover, asenapine is a partial agonist of the 5-HT_1A_ receptor [33,34,35]. Asenapine is primarily metabolized by CYP1A2 and to a lesser extent by CYP3A4 and CYP2D6 [34].

As mentioned above, asenapine exhibits a broad pharmacological profile that targets a wide range of neurotransmitter receptors with variable affinities. As a result, the neuroleptic increases dopamine, norepinephrine and acetylcholine efflux in cortical and limbic brain areas [36]. Asenapine also potentiates cortical glutamatergic neurotransmission and shows antipsychotic, antidepressant and pro-cognitive activities in animal behavioral models. Chronic treatment with asenapine affects the number of dopamine, serotonin, glutamate, adrenergic and cholinergic receptor subtypes in different brain areas [37]. These pharmacological properties of asenapine may contribute to its unique therapeutic profile in the treatment of schizophrenia and other psychiatric disorders.

Antipsychotics are used as monotherapy, as well as adjunctive or augmentation therapy in a number of psychiatric disorders. Concomitant therapy with antipsychotics may result in pharmacokinetic interactions producing adverse reactions. Due to their large volume of distribution, lipophilicity and extensive protein binding, the metabolic clearance of most antipsychotics is remarkably slow [38]. Therefore, drug interactions between psychotropic drugs can result in poor tolerability and/or reduced efficacy impacting the clinical outcomes of patients. Certain medications exhibit characteristics which also make them prone to drug–drug interactions, especially those with narrow therapeutic windows, non-linear pharmacokinetics and long half-lives [39]. The majority of clinically-relevant pharmacokinetic drug–drug interactions with antipsychotics occur as a consequence of drug-induced changes in hepatic metabolism, through the inhibition or induction of CYP enzymes [40,41]. When co-administered with inducers or inhibitors of CYP enzymes, plasma levels of antipsychotics may be reduced or increased, respectively, resulting in a diminished effectiveness of an antipsychotic or an increased risk of adverse events [42]. Similar effects may be observed with other drugs, when they are combined with antipsychotics [43,44] including asenapine [45,46], as suggested by our recent in vitro studies with human liver microsomes and human hepatocytes.

Schizophrenia and bipolar disorders are chronic illnesses requiring long-term therapy. Our recent in vitro studies have shown that asenapine directly inhibits the CYP enzymes CYP1A2 and CYP2D6 in liver microsomes via a mixed or competitive mechanism, respectively, and decreases the CYP1A2 expression in human hepatocyte culture [45,46]. However, the effect of prolonged asenapine treatment on liver cytochrome P450 expression in vivo has not been studied as yet, though its broad spectrum of target receptors suggests a possibility of interference with neuroendocrine regulation of physiological processes by this neuroleptic. Since comorbidity and concomitant medications are common in psychiatric patients, pharmacokinetic interactions between psychotropic drugs at the level of cytochrome P450 may occur. Therefore, the aim of our present research was to investigate the effect of two-week treatment with asenapine on the expression and activity of cytochrome P450 in rat liver.

## 2. Results

### 2.1. The Effect of Two-Week Treatment with Asenapine on the CYP Activity in Rat Liver Microsomes

**Figure 1:**
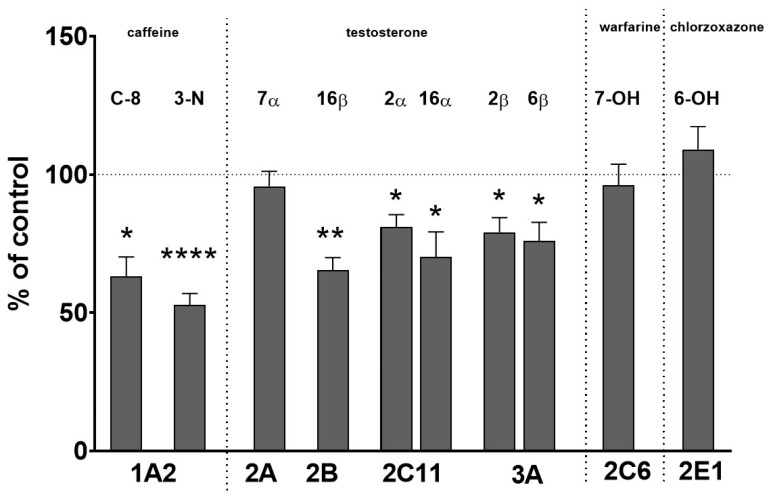
The effect of the chronic treatment of asenapine on activities of different cytochrome P450 enzymes, measured as the rates of CYP-specific reactions in rat liver microsomes: caffeine 8-hydroxylation and 3-N-demethylation (CYP1A), testosterone 7α- (CYP2A), 16β- (CYP2B), 2α- and 16α- (CYP2C11), and 2β- and 6β- (CYP3A) hydroxylation, warfarin 7-hydroxylation (CYP2C6) and chlorzoxazone 6-hydroksylation (CYP2E1). All values are shown as the mean ± S.E.M. (n = 12). Statistical significance was assessed by Student’s *t*-test and marked as * *p* < 0.05; ** *p* < 0.01; **** *p* < 0.0001, compared to the control. The control values (picomoles per milligram protein per minute) are as follows: 10.70 ± 2.84 (8-hydroxycaffeine), 1.57 ± 0.61 (3-N-demethylcaffeine); 107.5 ± 23.3, 39.8 ± 9.4, 650.5 ± 123.8, 363.4 ± 25.5, 30.9 ± 7.1, and 843.2 ± 257.2 (7α-, 16β-, 2α-, 16α-, 2β-, and 6β-hydroxytestosterone, respectively), 12.9 ± 3.5 (7-hydroxywarfarin) and 3.35 ± 0.8 (nmol/mg protein/min, 6-hydroxychlorzoxazone).

The obtained results indicate that, during long-term treatment with asenapine, a significant decrease in the activity of CYP1A, CYP2B, CYP2C11 and CYP3A was observed, but the activity of CYP2C6, CYP2A and CYP2E1 remained unchanged (Figure 1).

The activity of CYP1A, measured as the caffeine C-8-hydroxylation and 3-N-demetylation, decreased to 63% and 52% of the control, respectively. Asenapine decreased the CYP2B activity measured as the testosterone 16β-hydroxylation to 65% of the control. The CYP2C11 activity, estimated as the rate of the 2α- or 16α-hydroxylation of testosterone declined to 81% and 70% of the control, respectively. The activity of CYP3A, assessed as the rate of the 2β- or 6β-hydroxylation of testosterone dropped down to 79% and 76% of the control, respectively. The rate of warfarin 7-hydroxylation, corresponding to the CYP2C6 activity, the rate of testosterone 7α-hydroxylation representing the CYP2A activity and the rate of chlorzoxazone 6-hydroxylation, indicative of the CYP2E1 activity, were not significantly affected by asenapine (Figure 1).

### 2.2. The Influence of Two-Week Treatment with Asenapine on the CYP Protein Level in Rat Liver Microsomes

**Figure 2:**
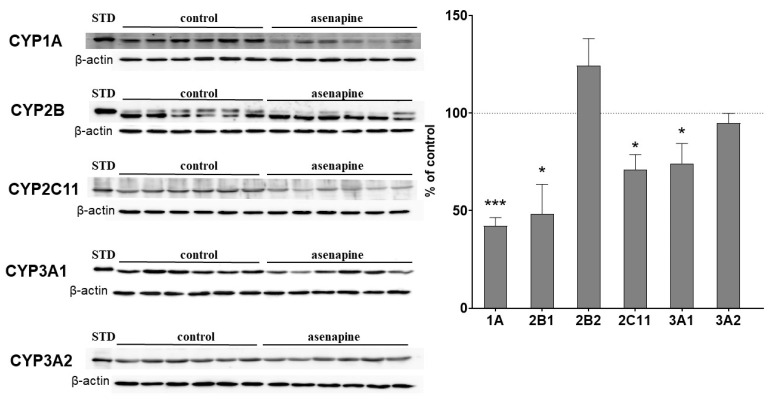
The effect of chronic asenapine treatment on the protein level of CYP1A, CYP2B1, CYP2B2, CYP2C11, CYP3A1 and CYP3A2 enzymes in rat liver microsomes. Microsomal proteins, 10 μg, were subjected to the Western immunoblot analysis. The results are presented as representative blots from six (for the control and for asenapine treatment) separate rats per treatment. The data are expressed as the mean ± SEM (*n* = 12). Statistical significance was assessed by Student’s *t*-test and marked as * *p* < 0.05, *** *p* < 0.001, compared to the control.

The protein level of CYP enzymes was measured by Western blotting in the liver microsomes from asenapine-treated rats. The decreased CYP activity correlated positively with the diminished level of the enzyme protein after administration of the studied drug. Asenapine significantly decreased the CYP1A protein to 42% of the control. The neuroleptic diminished the CYP2B1 protein level (but not that of CYP2B2) to 48% of the control. The CYP2C11 protein level was lowered by asenapine to 71% of the control. The CYP3A1 protein level was reduced by the neuroleptic to 74% of the control, while the amount of CYP3A2 protein remained unaltered (Figure 2).

### 2.3. The Effect of Two-Week Treatment with Asenapine on CYP Gene Expression in the Liver

**Figure 3:**
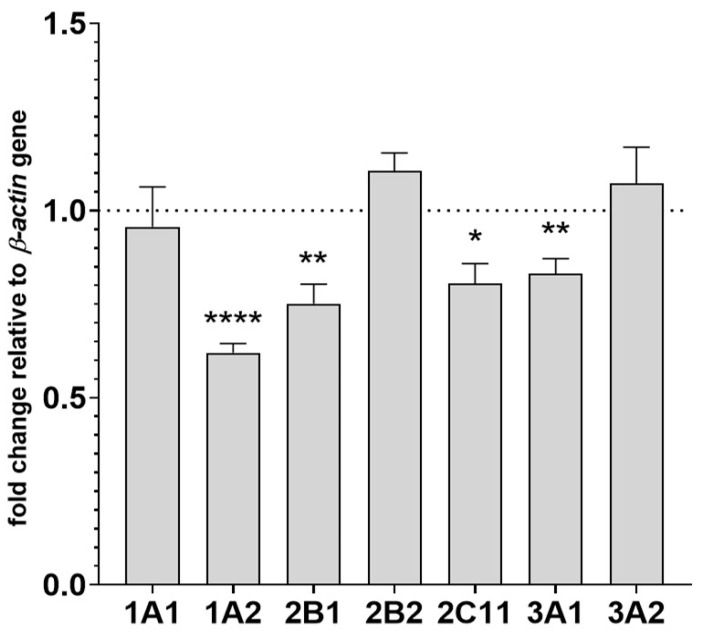
The effect of chronic treatment with asenapine on the mRNA levels of CYP1A1, CYP1A2, CYP2B1, CYP2B2, CYP2C11, CYP3A1 and CYP3A2 genes in the liver. The results are expressed as the fold-change in relation to the *beta-actin* housekeeping gene. All the values are the mean fold-change calculated by the comparative delta-delta Ct method for the control and asenapine-treated groups. All values are the means ± S.E.M. (*n* = 12). The results were calculated using Student’s *t*-test. Statistical significance is shown as * *p* < 0.05, ** *p* < 0.01, **** *p* < 0.0001 vs. control group.

The mRNA levels of CYP genes were measured in the liver tissue to further investigate the molecular mechanism of the observed changes in CYP activity and protein levels. The decreased activities and protein levels of CYP1A, CYP2B, CYP2C11 and CYP3A positively correlated with the changes in CYP mRNA level after administration of asenapine. After two-week treatment with the tested neuroleptic, decreased levels of CYP1A2, CYP2B1, CYP2C11 and CYP3A1 mRNAs were found (to 62%, 75%, 80% and 83% of the control, respectively). The examined drug did not produce any significant changes in the mRNA levels of CYP1A1, CYP2B2 and CYP3A2 genes (Figure 3).

### 2.4. The Effect of Two-Week Treatment with Asenapine on the Pituitary GHRH and Serum Concentrations of Hormones and Cytokines

**Figure 4:**
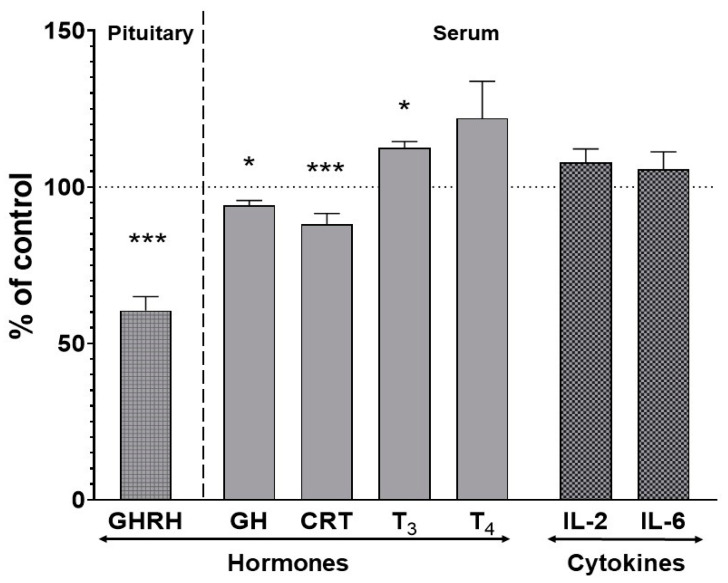
The effect of chronic asenapine treatment on the levels of pituitary and serum hormones and serum cytokine concentrations. All the values are the mean ± S.E.M. of the control (*n* = 12) and asenapine treatment (*n* = 12) group. Statistical significance was assessed by Student’s *t*-test and shown as * *p* < 0.05 or *** *p* < 0.001 compared to the control. The absolute control values were: 38.09 ± 4.47 ng/mg for pituitary growth hormone-releasing hormone (GHRH); 5.5 ± 0.31 ng/mL, 16.46 ± 1.62 ng/mL, 1.27 ± 0.094 ng/mL, 3.2 ± 0.97 ng/mL, 60.15 ± 8.43 ng/mL and 1.0 ± 0.18 ng/mL for serum growth hormone (GH), corticosterone (CRT), triiodothyronine (T_3_), thyroxine (T_4_), interleukin-2 (IL-2) and interleukin-6 (IL-6), respectively.

The level of growth hormone-releasing hormone (GHRH) in the pituitary gland decreased to 60% of the control after chronic treatment with asenapine. Serum concentrations of growth hormone (GH), corticosterone (CRT) and thyroid hormones (T_3_ and T_4_) that directly regulate cytochrome P450 expression were also affected by asenapine. The ELISA test revealed a significant decrease in the serum concentration of the corticosterone and growth hormone (to 88% and 94% of the control, respectively) and an increase in the thyroid hormone triiodothyronine (T_3_) (to 112% of the control). The concentration of thyroxine (T_4_) was not significantly changed after chronic asenapine treatment (Figure 4).

No changes in the serum concentration of the investigated pro-inflammatory interleukins (IL-2 and IL-6) were observed after chronic asenapine treatment (Figure 4).

## 3. Discussion

This is the first report showing changes in the cytochrome P450 expression and function produced in vivo by prolonged asenapine treatment in the rat. In this study, we demonstrate the effect of the asenapine on the key cytochrome P450 enzymes, namely CYP1A, CYP2A, CYP2B, CYP2C, CYP2E1 and CYP3A. The obtained results indicate that chronic administration of the novel neuroleptic drug asenapine produces broad changes in the expression and activity of hormone-dependent cytochrome P450 enzymes in the liver.

Our study has shown that chronic administration of asenapine significantly diminishes the activity of CYP1A enzyme in rat liver, which positively correlates with the decreased protein and mRNA levels of the CYP1A2 enzyme. These results are consistent with our previous in vitro experiment using human hepatocytes, in which asenapine at therapeutic concentrations decreased the expression and activity of CYP1A2 [45]. However, the contribution of additional mechanisms to CYP1A2 regulation, produced by chronic treatment, cannot be excluded, since the effect found in the present in vivo experiment is more pronounced than that observed in vitro [45]. Moreover, the inhibitory effect of asenapine on CYP1A2 may proceed also by direct inhibition of CYP protein activity (binding to the enzyme), as shown in our previous in vitro study with human cDNA-expressed CYP enzymes and human liver microsomes, where asenapine potently inhibited CYP1A2 via a mixed mechanism [46].

Asenapine influences also other CYP enzymes examined in this work, namely CYP2B, CYP2C11 and CYP3A. The decreased metabolic activities of those CYPs correspond well with the results from the Western blot, where the amounts of CYP2B1, CYP2C11 and CYP3A1 proteins were decreased in the rat liver microsomes by asenapine treatment. Simultaneously, the CYP2B1, CYP2C11 and CYP3A1 mRNA levels dropped down, which indicates that negative regulation of CYP2B1, CYP2C11 and CYP3A1 enzyme expression by asenapine proceeds at a transcriptional level. On the other hand, asenapine did not affect the activity of CYP2A, CYP2C6 and CYP2E1, which are less susceptible to hormonal regulation (discussed in [16]). It is worth noting that, in our previous study with human hepatocytes, asenapine did not affect the expression and activity of CYP3A4 [45], which suggests the involvement of neuroendocrine mechanisms in the present in vivo experiment.

The obtained results are in line with earlier observations concerning the effects of neuroleptic drugs on cytochrome P450 after prolonged administration. Rane et al. [47] reported that sulpiride and remoxipride reduced the CYP3A expression/activity, while Wójcikowski et al. [48] showed that thioridazine lowered the CYP3A activity and protein level. In another study, Haduch et al. [49] reported that thioridazine, perazine and levomepromazine diminished the CYP2C11 activity and protein level. Recently, Kot et al. [18] demonstrated that 5-week treatment with lurasidone reduced the activity and protein level of CYP2B and CYP2C11.

The above-presented results suggest a common mechanism of cytochrome P450 regulation by the investigated neuroleptics. Thus, liver cytochrome P450 enzymes are regulated hormonally by pituitary growth hormone (GH) production, adrenal corticosterone (CRT) and thyroid hormones (T_3_ and T_4_). Hormones, by acting on their receptors (GHR, GR, TR, respectively) activate multiple signaling pathways, thus interacting (directly or indirectly) with different transcription factors and nuclear receptors (e.g., the arylhydrocarbon receptor AhR, the glucocorticoid receptor GR, the constitutive androstane receptor CAR and the pregnane X receptor PXR), which are involved in the regulation of CYP genes’ expression [50,51].

GH controls the expression of the main male rat isoform CYP2C11 and plays an important role in the regulation of CYP3A gene transcription [52,53]. Corticosterone is the main positive controller of CYP3A, and also affects the expression of CYP1A and CYP2B [54,55,56,57], whereas thyroid hormones have a negative influence on the expression of various CYP isoforms [58,59,60]. Chronic asenapine treatment leads to a diminished growth hormone-releasing hormone (GHRH), GH and corticosterone secretion and, in consequence, to the reduced expression and activity of CYP2C11, CYP3A, CYP2B and CYP1A enzymes. The CYP1A genes are transcriptionally governed by AhR, which is positively modulated by the physiological concentrations of glucocorticoids in the rat [56]. The signaling pathways of AhR and TR share several co-activators and transcription factors (e.g., retinoid X receptor), which may lead to a transcriptional cross-talk between these pathways and alterations in CYP1A gene expression [61].

**Figure 5:**
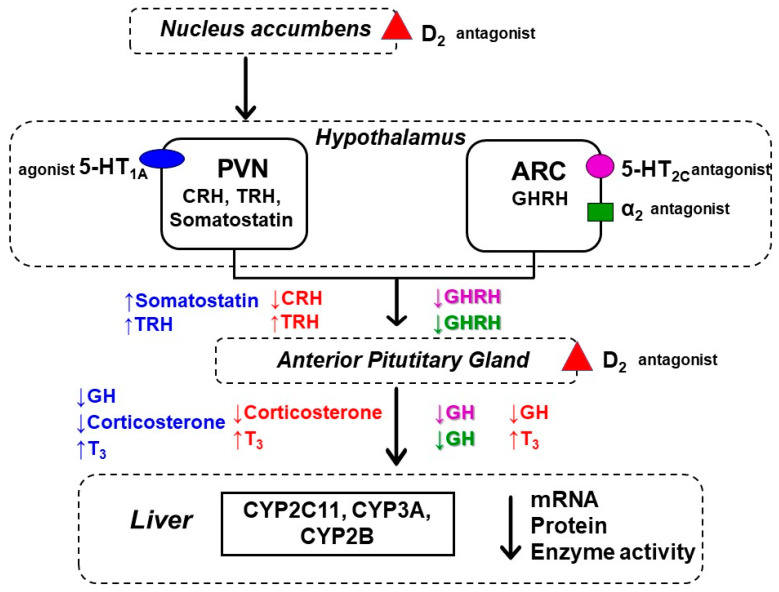
The central neuroendocrine regulation of liver cytochrome P450 by asenapine (a hypothetical scheme). **The D_2_ receptor blockade in the pituitary** decreases GH release and increases TSH-thyroid hormone (T_3_) secretion, which leads to down-regulation of the hormone-dependent CYP2C11, CYP3A1 and CYP2B1 expression/activity in the liver. The nucleus accumbens of the mesolimbic system projects to the paraventricular nucleus (PVN) of the hypothalamus. **The D_2_ receptor blockade in the nucleus accumbens** decreases the CRH release and corticosterone secretion and increases TRH release and thyroid hormone (T_3_) secretion, which leads to down-regulation of the above-mentioned enzymes (based on Wójcikowski and Daniel [13,17]). **The activation of the 5-HT_1A_ receptors in the PVN** increases somatostatin and TRH release, which leads to decreased GH and corticosterone secretion and increased T_3_ secretion and, in turn, to a reduction in the hormone-dependent CYP2C11, CYP3A1 and CYP2B1 expression/activity in the liver (based on Bromek and Daniel [16]). **The 5-HT_2C_ receptor blockade in the arcuate nucleus (ARC)** of the hypothalamus inhibits GHRH release and thus pituitary GH secretion, which leads to decreased expression/activity of the GH-dependent CYP2C11 and CYP3A1 (based on Bromek and Daniel [16]). **The α_2_ receptor blockade in the ARC** inhibits the GHRH-GH hormonal pathway and down-regulates the GH-dependent expression/activity of CYP enzymes in the liver (based on Sadakierska-Chudy et al. [14] and Bromek et al. [16]). ARC, arcuate nucleus; GHRH, growth hormone-releasing hormone; GH, growth hormone; PVN, the paraventricular nucleus; CRH, corticotropin-releasing hormone; TRH, thyrotropin-releasing hormone.

As mentioned in the introduction, our previous studies provided direct evidence of an important role of the brain dopaminergic, serotonergic and noradrenergic systems in the neuroendocrine regulation of CYP expression in rat liver [13,14,16]. Accordingly, drugs blocking dopaminergic D_2_ receptors in the brain (in the pituitary and mesolimbic pathway) decrease the secretion of growth hormone and corticosterone and increase thyroid hormone secretion, which leads to a decrease in the expression of CYP genes, including CYP2B, CYP2C11, CYP3A [13]. Therefore, a possible cause of the inhibitory effects of asenapine on CYP2B, CYP2C11, CYP3A expression may lie in its ability to block dopaminergic D_2_ receptors in the brain and, in consequence, to influence secretion of CYP-regulating hormones (Figure 5).

On the other hand, asenapine may negatively regulate hormone-dependent CYP enzymes in the liver via brain serotonergic mechanisms. As a partial 5-HT_1A_ receptor agonist [38,62], asenapine can stimulate somatostatin secretion in the hypothalamus and in this way, it can decrease growth hormone and corticosterone concentration in the blood, which leads to a reduction in the expression and activity of the hormone-dependent CYP2C11 and CYP3A1 in the liver [16]. Furthermore, as a 5-HT_2C_ receptor antagonist [63], asenapine may suppress hypothalamic GHRH release and pituitary GH secretion, leading to a negative regulation of CYP2C11 and CYP3A enzymes [16]. In addition, blockade of adrenergic receptors by asenapine [33,34], may also affect the observed effect of the neuroleptic on cytochrome P450, since the brain noradrenergic system was shown to play an important role in the regulation of liver cytochrome P450 [14,15]. Besides, a direct inhibitory action of asenapine on adrenergic receptors present in the liver and on respective signaling pathways mediating CYP regulation is also possible [51].

Hence, three different mechanisms of asenapine effect on liver cytochrome P450 are possible during long-term treatment with asenapine (and with other neuroleptics): (1) a direct effect on cytochrome P450 (CYP1A2, CYP2D6), as a result of binding to the enzyme protein, as shown in our previous in vitro studies with liver microsomes [46]; (2) an inhibition of CYP gene expression in the liver (CYP1A2), as shown in human hepatocyte culture [45]; and (3) an impact of the drug on brain receptors and neuroendocrine pathways affecting physiological regulation of CYP enzymes’ expression, as shown in the present in vivo study (Figure 5). Thus, asenapine can produce drug–drug metabolic interactions involving different mechanisms and CYP enzymes during combined continuous therapy. Increased levels of concomitantly administered drugs (e.g., antidepressants) may be expected, which require adequate dosing adjustment.

In summary, two-week administration of asenapine at pharmacological dosage decreases pituitary GHRH level, serum GH concentration and hepatic GH-related CYP2C11 expression and activity. At the same time, asenapine decreases serum concentration of corticosterone and corticosterone-regulated CYP1A2, CYP2B1 and CYP3A1 expression and activity. Simultaneously, asenapine increases serum concentration of triiodothyronine, the hormone which negatively regulates the investigated CYP enzymes. The presented results indicate the contribution of neuroendocrine mechanisms to the regulation of liver cytochrome P450 by asenapine treatment, which cannot be observed in in vitro experimental models. They indicate that new neuroactive drugs should be tested also in vivo for their interaction with cytochrome P450, which enables the full spectrum discovery of their mechanisms of action on the enzyme.

## 4. Materials and Methods

### 4.1. Drugs and Chemicals

Asenapine maleate, NADP, NADPH, glucose-6-phosphate-dehydrogenase, glucose-6-phosphate, caffeine and its metabolites, bufuralol and its metabolite 1′-hydroxybufuralol, chlorzoxazone and its metabolite 6-hydroxychlorzoxazone and RNA-free water were purchased from Sigma (St. Louis, MO, USA). Steraloids (Newport, KY, USA) provided testosterone and its metabolites. Warfarin was donated by Merck (Darmstadt, Germany), and 7-hydroxywarfarin was synthesized at Maj Institute of Pharmacology, Kraków, Poland. The primary rabbit polyclonal anti-rat CYP1A1/2, CYP3A1 and CYP3A2 antibodies came from Millipore (Temecula, CA, USA), anti-rat CYP2C11 antibodies were obtained from Thermo Fisher Scientific (Walthman, MA, USA). The monoclonal mouse anti-rat CYP2B1/2B2 was form Santa Cruz Biotechnology (Dallas, TX, USA). The polyclonal anti-rat β-actin antibody was purchased from Sigma (St. Louis, MO, USA). Horseradish peroxidase-labeled secondary antibodies and goat anti-mouse antibodies were from Jackson ImmunoResearch (West Grove, PA, USA) and goat anti-rabbit antibodies were from Vector Laboratories (Burlingame, CA, USA). Rat cDNA-expressed CYP1A2, CYP2B1, CYP2C11, CYP3A1, CYP3A2 (Supersomes) were from Gentest Corp. (Woburn, MA, USA). The chemiluminescence reagents SuperSignal West Pico PLUS Chemiluminescent Substrate kit came from Thermo Fisher Scientific (Walthman, MA, USA). For RNA isolation, a Total RNA Mini kit purchased from A&A Biotechnology (Gdynia, Poland) was used. A High-Capacity cDNA Reverse Transcription Kit, TaqMan assays and the TaqMan Gene Expression Master Mix from Life Technologies (Carlsbad, CA, USA) were used for mRNA estimation. The ELISA kits for growth hormone, corticosterone, T_3_, T_4_, Il-2 and Il-6 (Bioassay Technology Laboratory, Shanghai, China) were used. The kit for growth hormone-releasing hormone (GHRH) came from MyBiosource (San Diego, CA, USA). All the organic solvents of HPLC purity were provided by Merck (Darmstadt, Germany).

### 4.2. Animal Procedure and Preparation of Liver Microsomes

Male Wistar Han rats (Charles River Laboratories, Sulzfeld, Germany), weighing 280–300 g, were housed with food and water freely available, and were maintained on a 12-h light/dark cycle (lights on at 08:00 h) under conditions of constant temperature (22 ± 2 °C) and humidity (50 ± 5%). All procedures used in this study were conducted in compliance with the rules and principles of the 86/609/EEC Directive and were approved by the Bioethical Committee of the Maj Institute of Pharmacology, Polish Academy of Sciences, Krakow, Poland. Rats (*n* = 12 for each treatment group) received subcutaneously (s.c.) asenapine (0.3 mg/kg dissolved in saline) or vehicle (saline) for two weeks. The administered dose was chosen on the basis of its activity in pharmacological, neurochemical and behavioral paradigms shown in previous studies in rats [62,63,64]. Rats were sacrificed at 24 h after the last dose. Livers were quickly isolated, frozen in dry ice and stored at −80 °C. The blood was collected, and the serum was separated by centrifugation and stored at −80 °C. Liver microsomes were prepared by differential centrifugation in 20 mM Tris/KCL buffer (pH = 7.4), including washing with 0.15 mM KCL according to the conventional method [48].

### 4.3. Determination of CYP Enzyme Activity in Liver Microsomes

In vitro studies into CYP-specific metabolism of caffeine, warfarin, chlorzoxazone and testosterone in liver microsomes were carried out at the linear dependence of product formation on time, protein and substrate concentration, in the previously optimized conditions. Incubations were conducted in a system containing liver microsomes (ca. 1 mg of protein/mL) and NADP or NADPH-generating system. The total protein level in liver microsomes was measured by the method of Lowry using bovine serum albumin as a standard [65]. The activity of CYP1A2 was determined by measuring the rate of caffeine metabolism, C-8-hydroxylation and 3-N-demethylation at a substrate concentration of 100 μM. The final incubation volume was 1 mL, incubation time was 50 min. Caffeine and its metabolites were analyzed by HPLC with UV detection [18]. The activity of CYP2C6 was studied by measuring the rate of warfarin 7-hydroxylation at a substrate concentration of 60 μM. The final incubation volume was 0.5 mL, incubation time was 15 min. Warfarin and its metabolite were analyzed by HPLC with fluorescence detection [18]. The activities of CYP2A, CYP2B, CYP2C11 and CYP3A were studied by measuring the rate of CYP-specific reactions: the 7α-, 16β-, 2α- and 16α-, 2β- and 6β-hydroxylation of testosterone, respectively, at a substrate concentration of 100 μM and incubation time 15 min. The final incubation volume was 1 mL. Testosterone and its metabolites were analyzed by HPLC with UV detection [48,49]. The activity of CYP2E1 was studied by measuring the rate of 6-hydroksylation of chlorzoxazone at a substance concentration of 200 μM. Briefly, incubations were carried out in a system containing liver microsomes (ca. 1 mg of protein/mL), a Tris/KCl buffer (50 mM, pH = 7.4), MgCl_2_ (3.0 mM), EDTA (1 mM), NADP (1.0 mM), glucose 6-phosphate (5 mM) and glucose-6-phosphate-dehydrogenase (1 U in 1 mL) at 37 °C. The final incubation volume was 0.5 mL. After a 20-min incubation, the reaction was stopped by adding 20 µL of HCl. The incubation samples were centrifuged at 17,000 g for 10 min and the aliquot was collected (350 μL). Chlorzoxazone and its metabolite were analyzed by MerckHitachi chromatograph “LaChrom” (Darmstadt, Germany), equipped with an UV detector. The analytical column (Supelcosil LC-18, 15 cm × 4.6 mm, 5 μm) was from Supelco (Bellefonte, PA, USA). The mobile phase consisted of water containing 0.05% (*v*/*v*) phosphoric acid and acetonitrile in the proportion 78:22 (*v*/*v*). The flow rate was 1.0 mL/min from 0–8 min, 1.5 mL/min from 8.1–14 min and 14.1–15 min from 1.0 mL/min. The analytical wavelength was 287 nm and the column temperature was 25 °C. Total analysis time was 15 min and the retention time of 6-hydroxychlorzoxazone and chlorzoxazone was 4.5 and 13.5 min, respectively [66,67]. The sensitivity of the method allowed for quantification of chlorzoxazone and 6-hydroxychlorzoxazone as low as 0.004 nmol and 0.003 nmol in one sample, respectively. The lower limit of quantification (LLOQ) was 0.009 nmol/mL for chlorzoxazone and 0.008 nmol/mL for 6-hydroxychlorzoxazone. The accuracy of the method amounted to 1.2%. The inter- and intra-assay coefficient of variance were about 6%.

### 4.4. An Analysis of CYP Proteins in Liver Microsomes

The protein levels of CYP1A, CYP2B1, CYP2B2, CYP2C11, CYP3A1 and CYP3A2 in the liver microsomes of control (*n* = 12) and chronically asenapine treated rats (*n* = 12) were estimated using Western immunoblot analyses. Ten micrograms of microsomal protein were separated on sodium dodecyl sulfate-polyacrylamide 4% stacking gel and 12% resolving gel, and then protein was electroblotted onto a nitrocellulose membrane. Primary monoclonal mouse anti-rat antibody raised against CYP2B1/2B2, polyclonal rabbit anti-rat antibody raised against CYP2C11, CYP1A, CYP3A1 and CYP3A2 were used. After incubation with primary antibody, the blots were incubated with secondary antibody, i.e., the appropriate species-specific horseradish peroxidase-conjugated anti-IgG. Rat cDNA-expressed CYP1A2, CYP2B1, CYP2C11 (5 μg), CYP3A1, CYP3A2 (1 μg) were used as respective standards. The intensity of the bands corresponding to the enzyme protein on a nitrocellulose membrane were measured with the Luminescent Image Analyzer LAS-1000 and quantified by the Image Reader LAS-1000 and Image Gauge 3.11 programs (Fuji Film, Tokyo, Japan). The obtained data were normalized to protein loading based on the β-actin levels.

### 4.5. CYP mRNA Expression Assay in Liver Tissue

The total RNA was isolated from frozen liver tissue using a Total RNA Mini kit following the manufacturer’s instructions. The quantity and quality of the isolated RNA were verified using a Synergy/HTX multi-mode reader (BioTek, Winoosk, VT, USA). The isolated RNA samples were stored at −20 °C for 24 h, until use. Total RNA (1 μg) was used to prepare cDNA by reverse transcription using a High-Capacity cDNA Reverse transcription Kit according to the manufacturer’s instructions. The expression of the genes encoding the cytochrome P450 enzymes CYP1A1 (Rn01418021_g1), CYP1A2 (Rn00561082_m1), (CYP2B1 (Rn01457880_m1), CYP2B2 (Rn02786833_m1), CYP2C11 (Rn01502203_m1), CYP3A1 (Rn03062228_m1), CYP3A2 (Rn00756461_m1) and for references gene encoding beta-actin ACTB (Rn00667869_m1) were detected by a real-time polymerase chain reaction (PCR) using TaqMan Gene Expression Master Mix and species-specific TaqMan type probes and primers (TaqMan Gene Expression Assay, Life Technologies). Real-time PCR runs were performed using the Bio-Rad CFX96 PCR system (Bio-Rad, Hercules, CA, USA). Gene expression was determined using 2-delta Ct method using β-actin expression as a reference, as described previously [18].

### 4.6. An Analysis of Hormones and Cytokines in the Pituitary and Blood Serum

The levels of pituitary GHRH and serum concentrations of hormones (GH, corticosterone, T_3_ and T_4_) and cytokines (IL-2 and IL-6) of control (*n* = 12) and asenapine-treated rats (*n* = 12) were measured using ELISA kits following the manufacturer’s instructions. Absorbance was measured using a Synergy/HTX multi-mode reader (BioTek, Winoosk, VT, USA).

### 4.7. Data Analysis

The obtained results are presented as the mean ± S.E.M. The statistical significance of changes in enzyme activity, protein level or gene expression was calculated using Student’s *t*-test compared to the control value, using GraphPad Prism 8.0 (GraphPad Prism Software Inc., San Diego, CA, USA). The results were regarded as statistically significant when *p* < 0.05.

## 5. Conclusions

The presented results indicate that chronic treatment with asenapine down-regulates cytochrome P450 and may slow the metabolism of CYP1A, CYP2B, CYP2C11 and CYP3A substrates (steroids and/or drugs). The observed effects of asenapine on cytochrome P450 may be related to its pharmacological action on dopaminergic, adrenergic and serotonergic receptors (in particular on D_2_, 5-HT_1A_, 5-HT_2C_ and α_2_ receptors), which affects the endocrine regulation of liver CYPs and the signaling pathways mediating enzyme expression in the liver. Hence, it seems conceivable that psychotropic drugs can affect liver cytochrome P450 via neuroendocrine regulation which, in this way, may lead to metabolic drug–drug interactions. Since asenapine is metabolized by CYP1A2, CYP2D and CYP3A, it is also possible that this drug may inhibit its own metabolism, thus the plasma concentration of asenapine in patients after prolonged treatment may be higher than expected based on a single dose. The obtained results also indicate the necessity of testing new neuroactive drugs for their interaction with cytochrome P450 not only in vitro, but also in vivo, which enables the observation of the full spectrum of their mechanisms of action on cytochrome P450 expression and activity, taking place in the brain and peripheral organs (e.g., the liver), including the neuroendocrine regulation of the enzyme.

## Data Availability

Data is contained within the article.
